# Structural and functional effects of acoustic exposure in goldfish: evidence for tonotopy in the teleost saccule

**DOI:** 10.1186/1471-2202-12-19

**Published:** 2011-02-15

**Authors:** Michael E Smith, Julie B Schuck, Ronald R Gilley, Brian D Rogers

**Affiliations:** 1 Department of Biology, Western Kentucky University, 1906 College Heights Blvd., Bowling Green, Kentucky 42101, USA

## Abstract

**Background:**

Mammalian and avian auditory hair cells display tonotopic mapping of frequency along the length of the cochlea and basilar papilla. It is not known whether the auditory hair cells of fishes possess a similar tonotopic organization in the saccule, which is thought to be the primary auditory receptor in teleosts. To investigate this question, we determined the location of hair cell damage in the saccules of goldfish (*Carassius auratus*) following exposure to specific frequencies. Subjects were divided into six groups of six fish each (five treatment groups plus control). The treatment groups were each exposed to one of five tones: 100, 400, 800, 2000, and 4000 Hz at 176 dB re 1 μPa root mean squared (RMS) for 48 hours. The saccules of each fish were dissected and labeled with phalloidin in order to visualize hair cell bundles. The hair cell bundles were counted at 19 specific locations in each saccule to determine the extent and location of hair cell damage. In addition to quantification of anatomical injury, hearing tests (using auditory evoked potentials) were performed on each fish immediately following sound exposure. Threshold shifts were calculated by subtracting control thresholds from post-sound exposure thresholds.

**Results:**

All sound-exposed fish exhibited significant hair cell and hearing loss following sound exposure. The location of hair cell loss varied along the length of the saccule in a graded manner with the frequency of sound exposure, with lower and higher frequencies damaging the more caudal and rostral regions of the saccule, respectively. Similarly, fish exposed to lower frequency tones exhibited greater threshold shifts at lower frequencies, while high-frequency tone exposure led to hearing loss at higher frequencies. In general, both hair cell and hearing loss declined as a function of increasing frequency of exposure tone, and there was a significant linear relationship between hair cell loss and hearing loss.

**Conclusions:**

The pattern of hair cell loss as a function of exposure tone frequency and saccular rostral-caudal location is similar to the pattern of hearing loss as a function of exposure tone frequency and hearing threshold frequency. This data suggest that the frequency analysis ability of goldfish is at least partially driven by peripheral tonotopy in the saccule.

## Background

Frequency discrimination is the ability of a listener to discriminate between two pure tones that differ only in frequency. Frequency discrimination in fishes has been established from pyschophysical studies of a limited number of species [1], but has been of great theoretical interest since the early fish hearing experiments of von Frisch [2]. The reason for this interest is that the otolith organs of fishes lack obvious macromechanical frequency selective processes such as those found in the basilar membrane of the mammalian cochlea [3]. Thus, it is unclear exactly how fish are able to distinguish between frequencies.

Indeed, the basis of frequency discrimination in general has been debated for well over a century. The two main models of frequency discrimination are the place theory and temporal theory of hearing. The complex history of these theories is reviewed elsewhere [4]. In brief, the place theory states that the perception of pitch depends upon the location of vibrations along the sensory epithelia (e.g., basilar membrane in the cochlea of mammals). The temporal theory argues that the perception of pitch depends upon the temporal patterns with which auditory neurons respond to sound, since waveforms of stimuli are well represented by patterns of phase-locking in the auditory nerve [5]. In reality, both place and temporal cues may be processed differentially at various levels of the auditory system as the information passes from the sensory epithelia of the ear to the auditory nuclei of the brainstem, and on to the mid- and forebrain. It is currently unknown whether frequency discrimination in fishes is largely due to peripheral processes (hair cells and their associated primary afferent neurons) or central processes (e.g., brainstem and midbrain auditory nuclei).

Many vertebrate auditory systems have been shown to exhibit tonotopic mapping, which is an orderly arrangement of frequency response in the sensory organ. For example, amphibians, reptiles, birds, and mammals are known to use tonotopic mapping to peripherally discriminate frequencies [6-9]. That is, sensitivity to specific frequencies varies across the length of the auditory sensory epithelia. For example, frequency response in the mammalian cochlea and the avian cochlea and basilar papilla is organized in a graduated manner, with highest discernable frequencies stimulating hair cells in the basal end, lowest frequencies stimulating the apical end, and intermediate frequencies stimulating hair cells in a graded manner in between the two extremes [8,10,11]. Frogs have a three-part auditory system which includes a low-frequency vibration/sound detector (the sacculus), a low- to mid-frequency sound detector and discriminator (the amphibian papilla), and a high-frequency sound detector (the basilar papilla [12]). Tonotopy in the ears allows at least some frequency analysis to take place peripherally, outside of the central nervous system. Although tonotopic mapping has been well demonstrated in mammals, birds, and frogs, it has not been adequately investigated in fishes.

Fishes hear using an ear that is similar in many aspects to the inner ear of other vertebrates, and have similar capabilities in performing auditory functions such as discriminating between sounds of varying intensity, frequency, and temporal patterns as other vertebrates [1]. Indeed, there is good evidence that the ears of terrestrial vertebrates evolved from the ears of fishes [13]. The fish ear has three semicircular canals that respond to movement of the head and three sensory organs, the saccule, lagena, and utricle. One or more of these end organs serves as a sound detector, depending upon the species [14]. Each ear organ has a macula of sensory hair cells that are similar to those found in the ears of all other vertebrates.

Exposure to loud sounds can damage or destroy macular hair cells and induce hearing threshold shifts in fishes [15-20]. Sound exposure followed by examination of the inner ear has been used to ascertain which regions of the inner ear are sensitive to particular frequencies in birds and mammals [11,21-23]. For example, in chicks (*Gallus domesticus*) exposed to tones, higher frequencies produced a loss of short hair cells in the basal region of the basilar papillae, while lower frequencies produced a broader region of hair cell loss in the apical region [9,10,23]. Similarly, mammals exposed to tones exhibit outer hair cell loss that varies systematically along the cochlea as a function of exposure frequency [24-26].

In order to better understand the role of peripheral processes involved in frequency discrimination in fishes, we examined the tonotopic pattern of saccular hair cell damage in response to tone exposures in goldfish (*Carassius auratus*). We also tested hearing loss in the same fishes to understand the relationship between localized hair cell loss and hearing loss. This is the first study to attempt to correlate patterns of hair cell loss with frequency-specific hearing loss in fishes.

## Results

### Effects of tone exposure on the goldfish saccule

Only data from the 100, 800, 2000, and 4000 Hz exposures are presented here.

It was difficult to obtain consistent auditory evoked potential recordings from fish exposed to the 400 Hz tone, and the saccules from these fish had extensive damage that made hair cell bundle counts difficult and variable. It is unclear why damage would be more extensive at this frequency, but two possibilities are that this frequency is near the frequency of peak hearing sensitivity in goldfish and that the sound stimulus exhibited much stronger harmonics at 400 Hz than was found in the other exposure frequencies. Thus, we did not analyze the 400 Hz exposure data.

**Figure 1:**
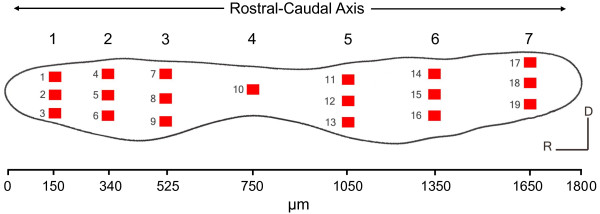
**Pre-selected locations on the goldfish saccule for hair cell quantification**. Schematic drawing of a left saccular epithelium from a goldfish, showing the 19 regions (50 μm × 50 μm each) in which phalloidin-labeled hair cell bundles were counted. Each column of boxes was represented as a rostral-caudal axis number from 1 to 7, with 1 and 7 being the most rostral and caudal, respectively. The corresponding distance (μm) from the rostral tip of the saccule for each rostral-caudal axis number is marked on the scale below the figure. Each row of boxes was represented as a dorsal-ventral axis number from 1 to 3, with 1 being dorsal and 3 being ventral.

**Figure 2:**
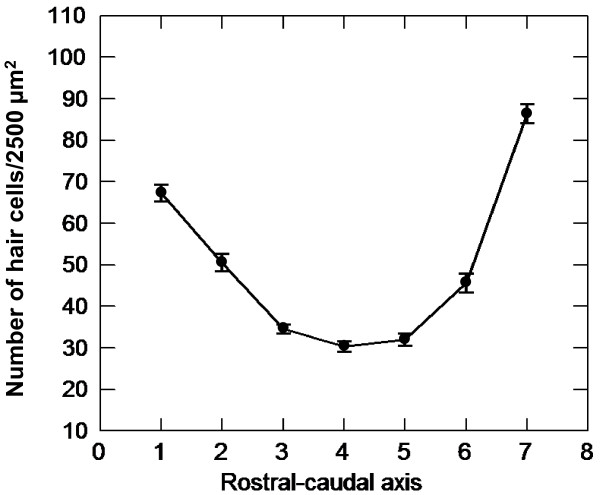
**Shift in hair cell density along the rostral-caudal axis of control goldfish saccules**. Mean (±S.E.) number of hair cell bundles/2500 μm^2 ^from control goldfish saccules as a function of location across the rostral-caudal axis of the saccule (n = 12).

Numbers of hair cell bundles were counted in 19 pre-selected 50 × 50 μm areas of both saccules of each fish (Figure 1). Among the control fish, hair cell bundle counts varied significantly along the length of the saccule (*P *< 0.001). Hair cell density was greatest at the far-rostral and far-caudal regions (approximately 70 and 90 hair cells/2500 μm^2^, respectively) and decreased toward the central region (approximately 30 hair cells/2500 μm^2^; Figure 2).

**Figure 3:**
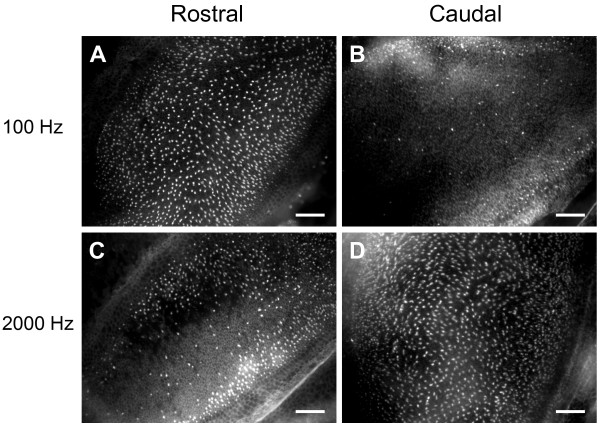
**Hair cell bundle loss as a function of tone frequency and saccule location**. Phalloidin-labeled saccular epithelia showing evidence of differential hair cell bundle loss between rostral (A, C) and caudal (B, D) regions in goldfish exposed to 100 (A, B) versus 2000 (C, D) Hz tones. Rostral areas photographed show locations 7-9 (rostral-caudal axis region 3). Caudal areas photographed show regions 11-13 (rostral-caudal axis region 5). Scale bars = 50 μm.

**Figure 4:**
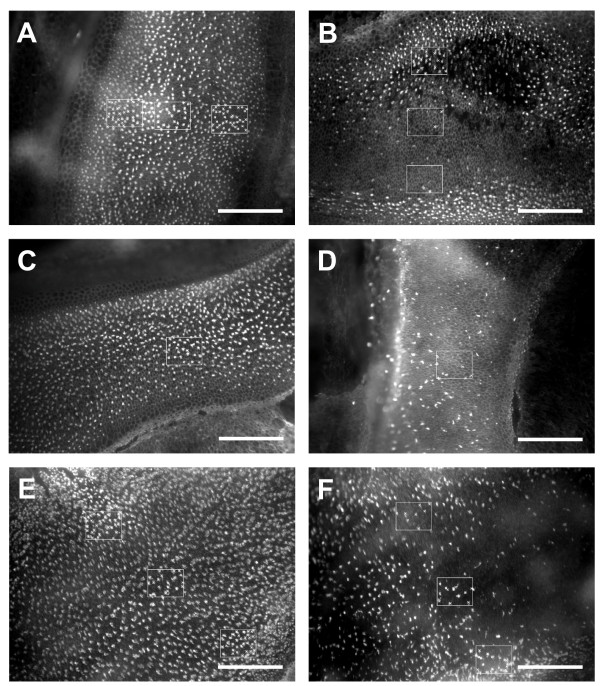
**Comparison between control and tone-exposed saccules at three different locations along the rostral-caudal axis**. Phalloidin-labeled saccular epithelia showing evidence of differential hair cell bundle loss between control (A, C, E) and tone-exposed (B, D, F = 2000, 800, 100 Hz exposures, respectively) saccules. A and B, C and D, and E and F show saccular locations 7-9, 10, and 14-16, respectively. 50 × 50 μm counting boxes are shown. X's were used to mark counted hair cell bundles. Scale bars = 100 μm.

**Figure 5:**
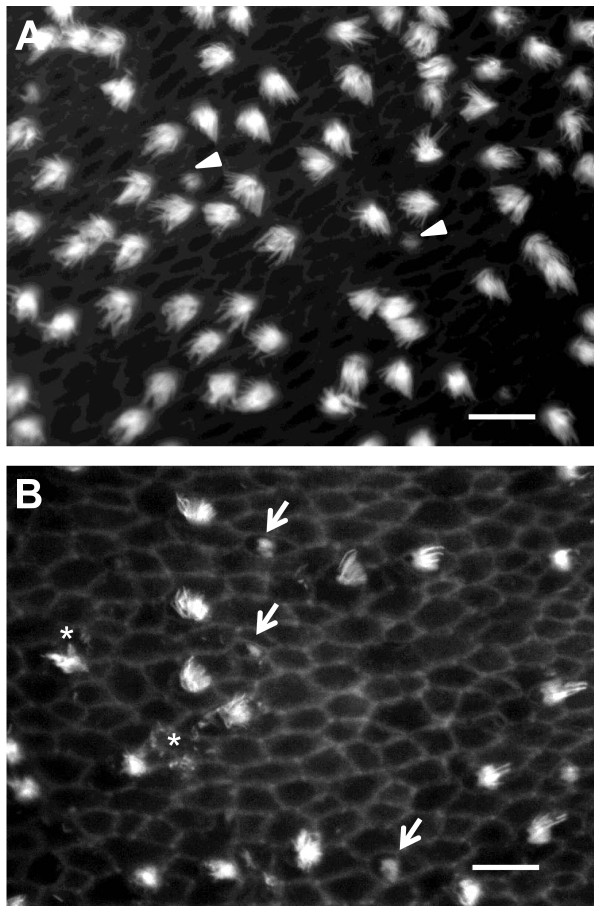
**Higher magnification comparison between control and tone-exposed saccular epithelia**. Phalloidin-labeled saccular epithelia viewed under a 100× objective showing evidence of differential hair cell bundle loss between control (A) and 4000 Hz tone-exposed (B) saccules at location 5. Arrow heads = presumed newly forming hair cell bundles. Arrows = bundleless hair cells. Asterisks = damaged stereocilia. Scale bars = 5 μm.

**Figure 6:**
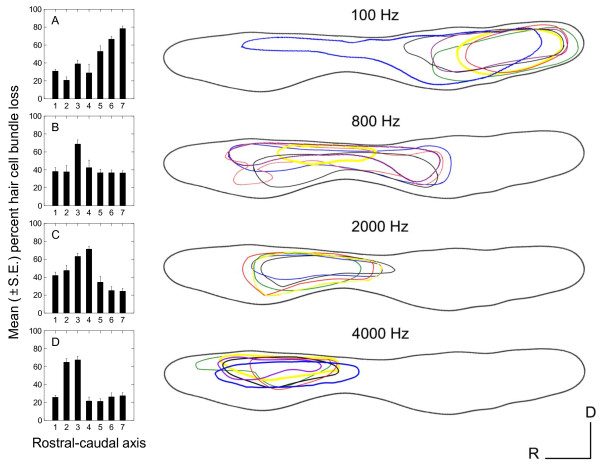
**Tonotopic distribution of tone-induced hair cell damage in the goldfish saccule**. Mean (±S.E.) percent hair cell loss as a function of location along the rostral-caudal axis of the saccule for each of the four tone exposures (A, B, C, D = 100, 800, 2000, and 4000 Hz, respectively). Region 1 represents the far rostral area of the saccule and region 7 represents the far caudal area (n = 10-12). Drawings of the distribution of damaged areas on the left saccular macula at the exposure tones indicated are to the right of the appropriate graph. Areas were marked as damaged if they appeared to be missing significant numbers of hair cells when viewed at low magnification (20× objective). Each colored line represents areas of hair cell bundle loss for an individual left saccule (n = 5-6). Similar patterns were found in right saccules.

All groups of sound-exposed fish exhibited significant loss of hair cell bundles (*P *< 0.001), but the pattern of hair cell bundle loss varied as a function of exposure tone frequency (*P *< 0.001) and rostral-caudal axis region (*P *< 0.001), with a significant interaction between these two variables (*P *< 0.001; Figures 3 and 4). The areas of loss were fairly localized. In the damaged areas, hair cell bundles were sparse. Some were completely missing, exposing the underlying cuticular plates, and some of the remaining hair cells exhibited ragged, splayed, and fractured stereocilia (Figure 5). Hair cell loss produced by the 4000 Hz tone occurred primarily in the rostral area, in regions 2 and 3. The 2000 Hz and 800 Hz tones also destroyed hair cells in the rostral area, but the general area of hair cell loss was shifted more caudally on the saccule. The 2000 Hz tone produced the greatest hair cell loss in rostral regions 3 and 4, and 800 Hz produced greatest loss in rostral region 3 (Figure 6). In general, areas of saccular hair cell loss were smaller for groups exposed to higher frequencies compared to areas of hair cell bundle loss in groups that were exposed to lower frequencies. The lowest frequency tone (100 Hz) only affected the caudal region of the saccule with the greatest hair cell loss occurring in regions 6 and 7 in most fish (Figure 6). The saccule from one individual exposed to the 100 Hz tone exhibited an aberrant pattern of damage that extended dorso-rostrally into region 3, but the whole epithelial region appeared to be damaged, not merely the hair cells.

Hair cell bundle loss varied across the width of the saccule as well, with the greatest loss occurring along the central portion of the saccule (*P *= 0.007; Figure 6). Differential loss across the width of the saccule was especially notable for the 4000 Hz group, in which the majority of hair cell loss occurred in the dorsal and central areas of the rostral region. The 100 Hz and 4000 Hz tones produced the greatest and smallest overall percentages of hair cell loss, respectively (Figure 6A and 6D).

### Effect of tone exposure on goldfish auditory thresholds

**Figure 7:**
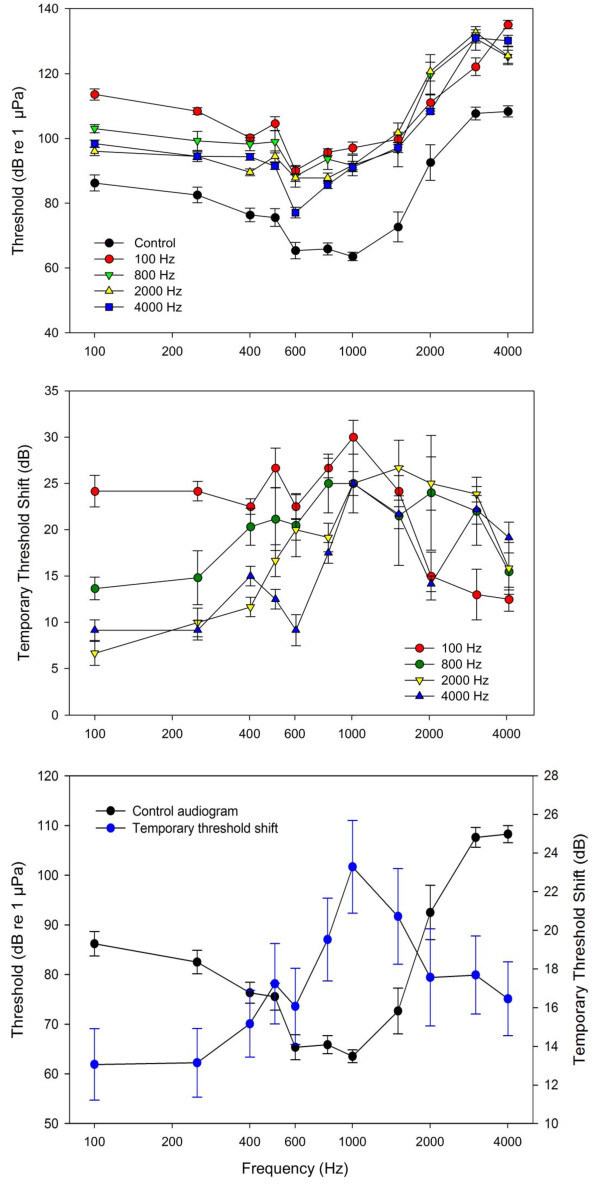
**Effect of tone frequency on auditory thresholds and temporary threshold shifts in goldfish**. (A) Auditory evoked potential (AEP) thresholds of control and experimental goldfish exposed to one of four tones. (B) Temporary threshold shift (TTS; calculated as threshold after sound exposure minus mean control levels) for the four tone exposures. (C) Control goldfish audiogram plotted with mean threshold shift averaged across all tone exposures to show that hearing loss was greatest at frequencies that goldfish are most sensitive (i.e., near 1 kHz). Values are means ± S.E; N = 6 per data point.

All sound-exposed fish had significantly higher hearing thresholds compared to controls (*P *< 0.001; Figure 7A). We will refer to these changes as temporary threshold shifts (TTS) since recovery from hearing loss has been reported for goldfish [17,20], and since permanent threshold shifts (PTS) have not been reported for fishes. Hearing loss varied with treatment, with mean TTS associated with 100 Hz tone exposure being greater than that of 2 and 4 kHz (*P *< 0.001; Figure 7B). Additionally, 800 Hz tone exposures resulted in threshold shifts that were greater than that of 4 kHz exposures (*P *= 0.007).

There were also significant differences in TTS between audiogram frequencies within specific tone exposures. For the 100 Hz tone exposure, TTS was significantly smaller at 2 - 4 kHz compared to lower frequencies (*P *< 0.01). No significant differences occurred in TTS across frequencies following the 800 Hz tone exposure although the highest mean TTS occurred at 800 and 1000 Hz (25 dB) and the lowest at the extremes of 100 Hz (13 dB) and 4 kHz (15 dB). In fish exposed to the 2 kHz tone, threshold shifts were greater at 1 - 2 kHz compared to 4 kHz and frequencies 500 Hz and lower (*P *< 0.05).

The 4 kHz tone resulted in perhaps the most complex pattern of hearing loss. TTS at 1, 1.5, 3, and 4 kHz, but not 2 kHz, were significantly higher than those at frequencies 600 Hz and lower (*P *< 0.05). In general, fish exposed to lower frequency tones exhibited greater threshold shifts at lower frequencies, while high-tone exposure led to greater hearing loss at higher frequencies (Figure 7B).

When TTS data for all treatments were pooled, there was a significant change in TTS across audiogram frequencies (*P *< 0.001), with maximal hearing loss at 1 kHz, the frequency of greatest hearing sensitivity in the goldfish (Figure 7C). *Post-hoc *Tukey tests showed that when TTS were pooled across tone treatments, TTS at 100 and 250 Hz were significantly lower than that of those at 800-1500 Hz (*P *< 0.005), TTS at 400 Hz was significantly lower than those of 800-1000 Hz (*P *< 0.02), and TTS at 1000 Hz was significantly higher than those of 600, 2000, and 4000 Hz (*P *< 0.02).

### Relationship between hair cell and hearing loss

**Figure 8:**
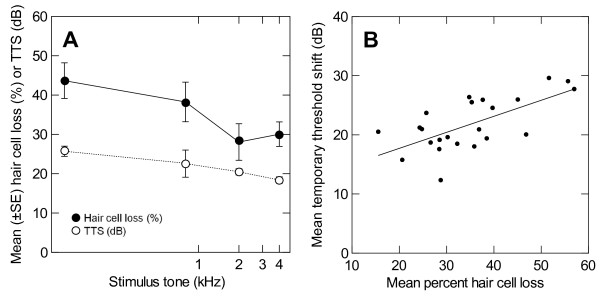
**Relationship between hair cell loss and temporary threshold shift in goldfish**. (A) Mean (±S.E.) percent hair cell loss (solid circles) and temporary threshold shift (TTS; open circles) as a function of exposure tone frequency. A decrease in both hair cell loss and hearing loss is seen with an increase in the frequency of acoustic exposure (N = 23). (B) Mean temporary threshold shift as a function of mean percent hair cell loss for individual goldfish (N = 23). Means are averaged across all saccule locations for hair cell loss and across all frequencies tested for threshold shifts.

To examine the relationship between hair cell loss and hearing loss more closely, mean percent hair cell loss (averaged across all saccular locations) and mean TTS (averaged across all hearing frequencies tested) were calculated for each individual fish, and plotted against the stimulus tone of the acoustic exposure. A similar pattern was evident for both hair cell and hearing loss - a decrease in loss with increased frequency of exposure (Figure 8A). When plotted together, a significant linear relationship exists between mean percent hair cell loss and mean TTS (P < 0.001; Figure 8B). This relationship predicts an 18 dB average hearing loss for a 20% average loss of hair cells, and a 29 dB average hearing loss for a 60% average loss of hair cells. Since the means in this analysis are averaged across both frequencies in the audiogram and locations of the saccule, no potential tonotopic relationship can be statistically tested without *a priori *subjectively assigning regions along the rostral-caudal axis of the saccule to frequencies of hearing sensitivity.

**Figure 9:**
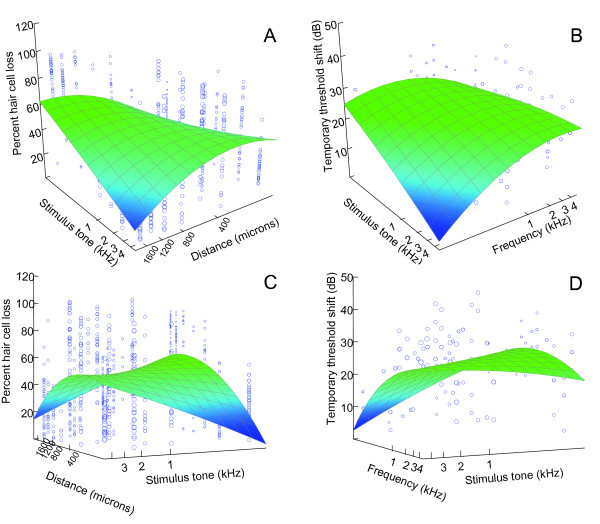
**Plots showing patterns of hair cell and hearing loss as a function of exposure frequency**. (A) Effects of tone exposure frequency and location along the rostral-caudal axis on percentage of hair cell bundle loss for each of the 19 predetermined locations for both left and right saccules of each fish (N = 819). Along the rostral-caudal axis, 1 (150 μm) is most rostral and 7 (1650 μm) is most caudal. (B) Effects of tone exposure frequency and hearing threshold frequency on hearing loss (threshold shifts) for each of the 11 hearing frequencies tested (N = 264). Plots (A) and (B) were rotated 180° to produce (C) and (D), respectively. Three-dimensional surfaces were constructed by distance-weighted least-squares smoothing of the original data, which imposes no *a priori *relationships between the variables.

In order to compare the relationship between specific locations of hair cell bundle loss and hearing loss at specific frequencies, we made two three-dimensional graphs. In the first graph, we plotted percent hair cell bundle loss at all 19 specified locations for each fish saccule as a function of frequency of the exposure tone and the location along the rostral-caudal axis of the saccule (Figure 9A). This graph reiterates the results previously discussed - that is, low frequency tone exposure resulted in dramatic hair cell loss in the caudal end of the saccule and minimal damage in the rostral side, while the opposite is true for high frequency tone exposures. In the second graph, we plotted hearing loss (TTS) for all fish tested as a function of frequency of the exposure tone and the frequency of the hearing threshold being tested (Figure 9B). A similar pattern was evident. Low frequency tone exposure resulted in significant threshold shifts at low frequencies, but minimal hearing loss at high frequencies, while high frequency tones resulted in significant threshold shifts at high frequencies, but not at lower frequencies.

While both plots result in smoothed surfaces that resemble a piece of paper with the opposite diagonal corners pulled down, one of the downward slopes is much more dramatic with the hair cell loss data (Figure 9C) than with the TTS data (Figure 9D). What this means is that at low frequencies of tone exposure, there is a dramatic drop in hair cell loss in the rostral saccule, but a relatively smaller drop in hearing loss at high frequencies. In other words, low frequency exposure tones still caused high-frequency TTS even though hair cell loss was fairly low in the rostral region which presumably detects high-frequency sounds.

The similarity between the smoothed surfaces of these two plots is what one would expect if the goldfish saccule is indeed tonotopically organized. Using these plots, and the tonotopic pattern of damage in the saccule (Figure 6), we suggest that along the rostral-caudal axis of the saccule, regions 1-3, 2-4, 3-5, and 6-7 correspond to frequency sensitivities near 4, 2, 0.8, and 0.1 kHz, respectively.

## Discussion

### Tonotopic damage in the goldfish saccule

We found a distinct caudal to rostral shift in hair cell bundle loss with exposure frequency, with the 100 Hz tone producing damage localized to the caudal saccule and the 4 kHz tone producing damage to the rostral saccule. A frequency-dependent spatial pattern of sound-induced damage in the inner ear has been reported for other vertebrates [9-11,21], but only one prior study has suggested that a similar tonotopic pattern may also be found in teleost fishes.

Enger [15] investigated the effect of intense tone exposure in Atlantic cod (*Gadus morhua*). Cod saccular maculae were examined for damage through scanning electron microscopy following exposure to intense tones (approximately 180 dB re 1 μPa) ranging in frequency from 50 to 400 Hz. Damaged areas of epithelia had an almost complete loss of hair cell stereocilia, leaving only cuticular plates or a few disorganized stereocilia on hair cells. As exposure frequency increased, the areas of damaged epithelia were located in successively more rostral regions of the saccule. It was also observed that all frequencies produced damage in the central region of the saccule.

Our data show a similar pattern of tone-induced hair cell loss in goldfish, despite differences in hearing sensitivity and bandwidth. The range of hearing is 50 to 470 Hz in cod, with hearing thresholds recorded at 75-80 dB re 1 μPa at 100 Hz [27], while goldfish hear a greater range of frequencies, from 20 to greater than 4000 Hz, with greatest sensitivity between 400 and 1000 Hz [1]. The Weberian ossicles of goldfish and other otophysan fishes mechanically link their swim bladder to their inner ear [28]. This specialization allows goldfish to be more sensitive to the pressure component of sound. Fish without such modifications, such as cod, are less sensitive to pressure, and possess hearing thresholds that are higher and cover narrower frequency bandwidths than fish with such specializations [29]. Since the ears of teleost fishes differ so much between species, it is likely that tonotopic patterns in the auditory epithelia also differ across taxa, especially between fishes that are pressure sensitive compared to fishes that detect only particle motion. Yet, the overall tonotopic pattern of auditory hair cell loss is similar between goldfish and cod, with low and high frequency tones damaging caudal and rostral portions of the saccule, respectively. This suggests that tonotopy of the saccule may function as a mechanism of peripheral frequency discrimination across multiple orders of fishes.

We found that hair cell loss occurred in the central region of the saccule for most tone-exposed goldfish (Figure 6) and that overall threshold shifts were greatest at middle frequencies (800-1500 Hz) for all tone-exposed fish (Figure 7C). Since these middle frequencies correspond to the frequencies of greatest sensitivity in the goldfish [17,18], we surmise that these centrally located hair cells preferentially respond to middle frequencies and that these hair cells are generally more prone to acoustically-induced damage. While this may be the result of morphological differences in these hair cells (e.g., kinocilia and stereocilia lengths), it is more likely the result of the mechanical connection between the sensory epithelium and the otolith, and the frequency-specific relative motion between the two.

Another pattern that was evident was that areas of hair cell damage became generally smaller with increasing exposure frequency. The frequency-position functions are logarithmic in nature in humans and other mammals [30,31]. That is, lower frequencies are represented by locations that are spread apart farther on the basilar membrane than higher frequencies, with the frequency-position distances decreasing with frequency. Although the fish ear does not have as precise of a frequency-place mechanism provided by the macromechanical arrangement of the cochlea, a similar logarithmic frequency-position function may occur in fishes. This would explain why there is little overlap in damaged areas of goldfish saccules caused by 100 and 800 Hz exposures (a difference of 700 Hz), and considerable overlap caused by 2000 and 4000 Hz exposures (a difference of 2000 Hz). In cod, there was a greater degree of overlap of damaged areas resulting from tone exposures ranging from 50 to 350 Hz [15]. This may be because the high intensity of sounds used (180 dB re 1 μPa) and/or the smaller bandwidth of hearing in cod (up to only 600 Hz [32]). Non-otophysan fishes have smaller bandwidths of hearing, poorer hearing sensitivity, and poorer frequency discrimination compared to otophysan fishes [1]. Thus, one might expect a coarser tonotopic map in non-otophysans compared to otophysan fishes like goldfish.

The high intensities used during the sound exposures in the current study may have been enough to spread the area of damage across the saccule beyond the region that normally responds to a given tone, affecting the sensitive, centrally-located hair cells. Previous studies have demonstrated that very intense sound damages an extended region of the hearing end organ in birds and mammals. For example, in chicks, the damaged area of the basilar papilla varies with exposure frequency, but also with tone intensity; a secondary region of damage is created with increased sound pressure level [11]. In the chick, exposure to lower frequencies produced a larger region of damage than did exposure to higher frequencies. This is similar to the data that we report here--in goldfish, the greatest hair cell loss occurred with the lowest stimulation frequency (100 Hz), and the greatest TTS also occurred at 100 Hz (Figure 8A).

Intense octave band noise can cause focal lesions that extend beyond the frequency-place range of the exposure sound in the cochlea of chinchillas [33,34], and it is known that the basilar membrane of mammals become more broadly tuned with increased stimulus intensity [35]. Whether such a pattern exists in goldfish is unknown. Future experiments with lower exposure intensities are needed to see if damaged areas become more localized as sound pressure level decreases.

### Potential mechanisms for tonotopy in the saccule

The mechanisms that contribute to tonotopic mapping in the fish saccule have not been fully elucidated, but there are several possibilities. One potential mechanism is that the otolith overlying the saccular epithelium vibrates differentially in response to varying frequency. While the modeling of fluid motion around otolith-like spheroids has recently been conducted [36], direct measurement of otolith vibration is rarely done. Sand and Michelsen [37] used laser vibrometry to measure the vertical vibration of saccular otoliths in perch (*Perca fluviatilis*) in response to four frequencies of horizontal movement. They found that the amplitude of vibration varied across the length of the otolith in a frequency-dependent manner, suggesting that the otolith motion had a rotational component and that this might explain observed peripheral frequency analysis in teleost fishes. The goldfish saccular otolith, the sagitta, is long, thin, and delicate with a twist in the middle similar to an airplane propeller [38]. It has been proposed that such an otolith design may have a role in frequency analysis [39], but little is known about the mechanical properties of the gelatinous otolithic membrane and its coupling with hair cells.

It is also likely that characteristics of the sensory cells themselves are at least partially responsible for the tonotopic damage found in this study. Our finding of a shift in hair cell density along the goldfish saccular rostral-caudal axis is consistent with that found by other researchers. Higgs et al. [40] found that hair cell density in zebrafish (*Danio rerio*), a close relative of goldfish, was greatest at the caudal tip, least dense in the middle, and intermediate at the rostral tip of the saccule. Similar patterns have also been found previously in goldfish [19,38,41].

Goldfish auditory hair cells also show ultrastructural differences that vary by location on the saccule [41]. For example, hair cell size, mitochondria and synaptic body sizes, subnuclear layering of the cisternae, and afferent diameter have also been found to vary across the length of the goldfish saccule [42]. In goldfish, hair cell stereociliar and kinociliar length varies by location, with longer kinocilia found in the caudal end and shorter kinocilia in the rostral end. We have also observed differences in stereociliar length across the length of the goldfish and zebrafish saccule (unpublished observations, Michael E. Smith). Interestingly, no such pattern of hair cell length is found in the nonotophysan kissing gourami *Helostoma temmincki *[43].

It is thought that fish hair cells demonstrate micromechanical tuning through differences in hair cell bundle stiffness, mode of attachment to the otolithic membrane [44], and electrical tuning [45,46]. Outer hair cell stereocilia length is strongly correlated with the frequency of best response in the mammalian cochlea, with shorter and longer hair cells responding best to higher and lower frequencies, respectively [47]. It appears that a similar arrangement is found in the goldfish saccule. Similarly, tonotopic gradients of mechanotransduction channel conductance have been found in hair cells of the hearing end organs of turtles, birds, and goldfish [48-51]. Hair cell resonance frequency gradients are also seen in turtles and frogs [52,53].

Goldfish possess excellent tone discrimination ability, distinguishing tone differences as small as 3.5 Hz at 50 Hz [54]. In addition to saccular tonotopy, which appears to be rather crude in fishes, other peripheral and central processes likely contribute to frequency analysis in fishes. Some frequency filtering occurs in goldfish at the saccular level through afferent nerve fibers. Fay and Ream [55] found four non-overlapping categories of saccular nerve fibers (untuned, low-frequency, mid-frequency and high-frequency). This data suggest that there are just a few filters across the hearing bandwidth and very broad tuning of primary auditory afferents in fishes [56-59]. Central processing appears to play a role in fine frequency discrimination through mechanisms such as phase-locking in auditory units in fish and amphibians, with the occurrence of phase-locking appearing to diminish along the auditory pathway [60]. For example, phase-locking has been found in the medulla of trout, cod, and goldfish [61-63], and in the torus semicircularis (TS) of trout and goldfish [61,64,65].

### Effect of tone frequency on goldfish threshold shifts

Goldfish threshold shifts varied with frequency of tone exposure as predicted, with the 100 Hz tone exposure causing greater threshold shifts at lower frequencies and 2000 and 4000 Hz tones causing threshold shifts primarily at higher frequencies. As there was significant overlap in regions of damaged saccular epithelia caused by different tone exposures, there was also considerable overlap in frequencies that were affected by a particular tone exposure. For example, the 100 Hz tone exposure caused significant threshold shifts across all frequencies but this effect was much greater at frequencies below 1.5 kHz, while the 4 kHz tone also caused significant threshold shifts across all frequencies but the largest threshold shifts occurred at frequencies ≥1 kHz (Figure 7B).

Such overlap has been seen in other studies in which fish were acoustically over-stimulated. For example, Popper and Clarke [66] found that goldfish that were exposed to an 800 Hz tone exhibited threshold shifts at both 500 and 800 Hz, although the threshold shifts were greater at 800 Hz. Similarly, fathead minnows (*Pimephales promelas*) exhibited an 8-11 dB TTS in response to 2 h of 142 dB re 1 μPa narrow bandwidth boat motor noise with a peak frequency near 1.3 kHz [67]. While the greatest threshold shift occurred at 1.5 kHz (near the peak frequency of the noise exposure), threshold shifts also occurred at 1 and 2 kHz. In previous studies, goldfish exposed to white noise exhibited hearing loss across their hearing bandwidth, with threshold shifts being greatest at 1 kHz, where hearing sensitivity is greatest [17,18]. Similarly, mean threshold shifts were generally greatest at 1 kHz in this study, even when the exposure tone varied considerably from 1 kHz (e.g., 100 Hz and 4 kHz; Figure 7C).

It is not surprising that the goldfish had the greatest hearing loss at frequencies where their hearing is most sensitive because the difference between the sound pressure level of the exposure tone and the level of hearing threshold is greatest at these frequencies. This concept was formalized as a predictive model of hearing loss called the linear threshold shift (LINTS) hypothesis [18]. In this model, threshold shifts increase linearly as a function of the sound pressure difference (SPD) between an exposure sound and baseline hearing thresholds, and not just the sound pressure level of the exposure sound *per se*. The advantage of using SPDs is that they account for differences in hearing sensitivity across different frequencies in the animal's audiogram. The LINTS model was developed using a white noise stimulus, with equal intensity across the frequencies tested. Since most organisms have U-shaped audiograms that are more sensitive at mid-frequencies and less sensitive at lower and higher frequencies, the SPD between a white noise stimulus and hearing thresholds is greatest at mid-frequencies. Thus, TTS is predicted to be greatest at these mid-frequencies [18].

The current study used tonal stimuli such that the SPD between the tone and the hearing threshold was greatest at the frequency of the exposure tone and should have been minimal at all other frequencies. Thus, according to the LINTS model, TTS would be expected only at or near the frequency of the exposure tone. This obviously did not occur-TTS occurred at multiple, if not all of the frequencies tested, although low frequency tones produced the greatest TTS at low frequencies and high frequency tones produced greatest TTS at high frequencies. We tested the LINTS relationship by plotting TTS against SPD and performing linear regression analysis between these two variables. In this analysis, only TTS at hearing frequencies equal to the exposure tone were used since the maximal sound pressure level measured was that of the exposure tone and not adjacent frequencies (e.g., TTS at 100 Hz as a result of the 100 Hz tone exposure). This relationship was not significant (TTS = 0.15 (SPD) + 13.3; R^2 ^= 0.13, P = 0.09). One potential explanation for this observation is that the stimulus intensity used in this study may have been too high for the LINTS model to make effective predictions. As previously discussed, louder exposure stimuli produce greater areas of sensory cell damage, and thus hearing loss, over a greater range of frequencies. A replication of this study at lower stimulation intensities would shed light on the overall validity of the LINTS model for predicting hearing loss in fishes.

### Relationship between hair cell and hearing loss

Loss of hair cells is correlated with a loss of hearing in other vertebrates [68,69] so we expected to find a similar relationship between hair cell bundle and hearing loss in fishes. Only one previous study has examined the relationship between hair cell loss and hearing loss in fishes. Smith et al. [20] exposed goldfish to white noise for 48 h, which resulted in both significant hair cell loss and threshold shifts. Within 7 days thresholds almost completely recovered, and this hearing recovery coincided with a regeneration of hair cells. Similar to the current study, TTS was greatest at 1 kHz, but hair cell loss was limited to the caudal and central regions of the saccule following the white noise exposure.

**Figure 10:**
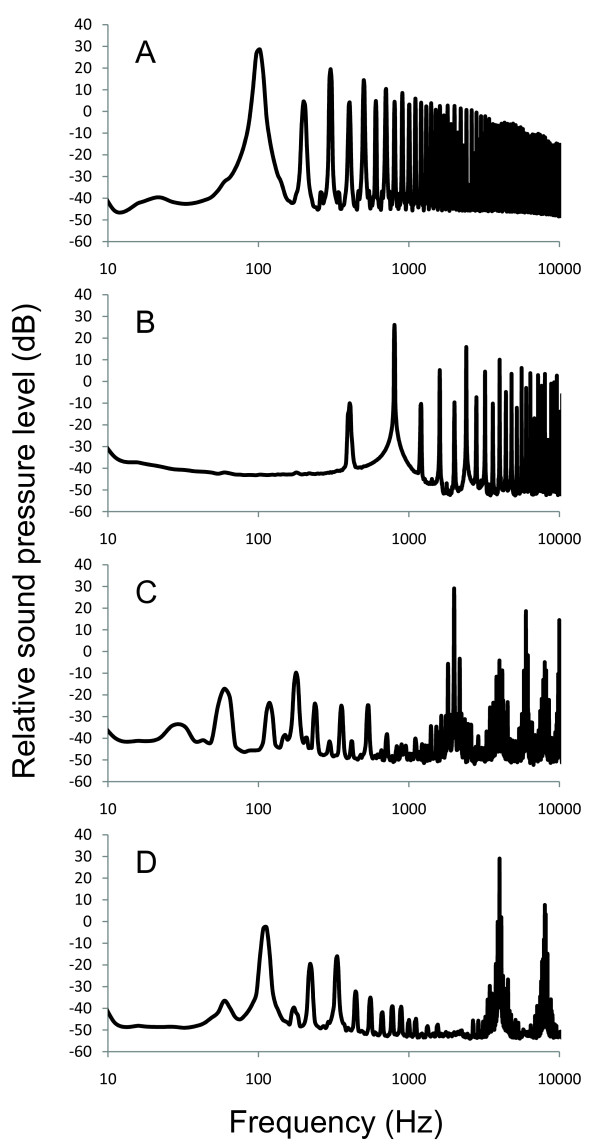
**Power spectra of the tones used for acoustic exposures**. Power spectra of the 100, 800, 2000, and 4000 Hz tones used for acoustic exposures of goldfish. Exposure tones were recorded 2 cm above the underwater speaker at a sound pressure level of 176 dB re 1 μPa RMS.

In the current study, there was considerable overlap in hearing loss across frequencies and hair cell loss across saccular regions for a given exposure tone. While this may be the result of the effect of the high intensity of the exposure stimuli that we used in this study, this overlap could also reflect the coarseness of the tonotopic map in the goldfish saccule, suggesting that the peripheral auditory filters are broadly tuned. Another possible explanation for this overlap is harmonics of our tone signals that were evident in our acoustic exposure and difficult to avoid in our small exposure chamber (Figure 10). While peak sound pressure levels at the nominal tone of interest were at least 10 dB greater than any harmonic, it is still possible that these harmonics of the nominal tone could have caused some auditory damage, thus broadening the area of saccular hair cell loss and the bandwidth of hearing loss.

A similar pattern was evident for both hair cell and hearing loss - a decrease in loss with increased frequency of exposure (Figure 8A). A similar relationship has been found in the chick basilar papilla, with lower frequency tone exposures producing greater hair cell loss over a greater area of the epithelium compared to damage from high frequency tones [11,69]. More narrow frequency bands of threshold shifts were associated with smaller regions of hair cell loss that resulted from higher frequency exposures. An opposite pattern is found in mammals, which are more sensitive at higher frequencies compared to birds and fishes. Guinea pigs exposed to pure tones exhibited much greater hair cell damage after exposure to frequencies of 1 kHz and above compared to that of 500 and 125 Hz [21]. Obviously, the shape of an organism's audiogram will have an effect on how susceptible its auditory system is to damage from a particular frequency of sound.

## Conclusions

We demonstrate that overexposure to pure tones causes tonotopically-organized morphological damage in the goldfish saccule. Damage is characterized as a significant decrease in hair cell bundle density. Areas of damage varied with tone frequency, with damage shifting rostrally across the length of the saccule as frequency increased. In addition, there was a corresponding shift in hearing loss with low and high frequency tones causing threshold shifts at lower and higher frequencies, respectively. These data suggest that goldfish use at least a crude level of tonotopy in the saccule to peripherally discriminate between tones.

## Methods

### Experimental animals

Goldfish (*Carassius auratus *L.) were chosen as model species because much is known about their hearing capabilities. They are otophysans and show sophisticated auditory capabilities including tone discrimination and auditory segregation [70-73]. Goldfish are also known to have sensitive hearing compared to many fishes, and thus are more susceptible to noise-induced hearing loss and damage to auditory hair cells [17,18,20].

Thirty-six fish ranging from 8-11 cm total length were obtained from Hunting Creek Fisheries (Thurmont, MD) and were housed in 170-L tanks of recirculated, filtered water maintained at approximately 25°C. All work was done under the supervision of the Institutional Animal Care and Use Committee of Western Kentucky University.

### Sound exposure

Subjects were divided into six groups of six fish each (five treatment groups plus control). The treatment groups were each exposed to one of five tones: 100, 400, 800, 2000, and 4000 Hz at 176 dB re 1 μPa root mean squared (RMS) played continuously for 48 hours. The tone stimuli were generated using a B&K Precision function generator (4017A) and played through an amplifier (5.3 Amp monoblock, Audiosource, San Francisco, CA, USA) to an underwater speaker (UW-30; University Sound Inc., Oklahoma City, OK, USA) placed centrally on the bottom of a 19-L cylindrical chamber. Sound pressure levels were measured via a Type 10CT hydrophone placed 2 cm directly above the center of the speaker and a Type 42AC Pistonphone calibrator (GRAS, Twinsburg, OH, USA). In order to maintain fish health during the 48 h exposure, fish were allowed to swim freely in the exposure chamber as in previous fish sound exposure experiments [17,18,20], but they spent a majority of their time towards the bottom of the chamber near the speaker. Thus, 176 dB re 1 μPa RMS represents a maximal exposure sound pressure level, with minimal SPLs in the chamber being approximately 10 dB lower. Exposure tones were digitally recorded as .wav files and imported into Audacity 1.2.6 (Verilogix, Inc.) to plot the power spectrum using a Hanning window and an FFT of 8192 (Figure 10). Immediately following sound exposure, the fish's hearing was tested by measuring auditory evoked potentials (AEP).

### Auditory evoked potentials (AEP)

AEP is a non-invasive method of measuring neural responses to auditory stimuli and is commonly used for measuring hearing in fishes and other vertebrates [74,75]. Each fish was anaesthetized with MS-222 (tricaine methanosulfonate), restrained in a mesh sling, and suspended under water in a 19-L plastic vessel (separate from the exposure chamber). Each fish was suspended so that the top of the head was approximately 6 cm below the surface of the water and 22 cm above the underwater speaker.

Stainless steel subdermal electrodes (27 ga, Rochester Electro-Medical, Inc., Tampa, FL) were used to record auditory evoked potentials. A reference electrode was inserted approximately 2 mm subdermally into the medial dorsal surface of the head between the anterior portion of the eyes while a recording electrode was placed 2 mm into the dorsal midline surface of the fish approximately halfway between the anterior insertion of the dorsal fin and the posterior edge of the operculae, directly over the brainstem. A ground electrode was placed in the tail musculature of the fish.

Sound stimuli were presented and AEP waveforms collected using SigGen and BioSig software running on a TDT physiology apparatus (Tucker-Davis Technologies Inc., Alachua, FL, USA). Sounds were computer generated via TDT software and passed through a Hafler P1000 power amplifier (Hafler, Tempe, AZ) connected to an underwater speaker (University Sound UW-30). Tone bursts were 15, 10, and 5 ms in total duration for 0.1 and 0.25, 4 and 6, and 0.8-4 kHz tones, respectively. Each tone pip had a 2 ms rise and fall time and were gated through a Hanning window (similar to the conditions of other AEP studies [17,18,20]). Responses to each tone burst at each SPL were collected using the BioSig software package, with 1000 responses averaged for each presentation. Auditory thresholds were determined at 11 frequencies for each fish (0.1, 0.25, 0.4, 0.5, 0.6, 0.8, 1, 1.5, 2, 3, and 4 kHz). The SPLs of each presented frequency were confirmed using a calibrated underwater hydrophone (calibration sensitivity of -195 dB re 1 V/μPa; ± 3 dB, 0.02-10 kHz, omnidirectional, GRAS Type 10CT, Denmark), placed in the same location where fish were held during AEP recording. Auditory thresholds were determined by visual inspection of auditory evoked potentials as has been done in previous studies [17,18].

### Characterization of hair cell bundle loss

Following AEP, subjects were euthanized by overdose with tricaine methansulfonate (MS-222). The heads were removed, injected with 4% paraformaldehyde, and placed in 4% paraformaldahyde overnight at 4°C. The heads were washed in 0.1 M phosphate buffer and the inner ears removed under a dissecting microscope. Both right and left saccules were then trimmed and incubated for 30 minutes in Alexa Fluor 488 phalloidin (Molecular Probes/Invitrogen, Carlsbad, CA, USA), which stains actin in hair cell bundles and cuticular plates. Saccules were mounted whole under a cover slip with Prolong Gold antifade reagent with 4',6-diamidino-2-phenylindole (DAPI) to stain nuclei.

Images of the saccule were taken with a 20× objective using a Zeiss Axioplan 2 (Germany) epifluorescent microscope and a Zeiss MRm digital CCD camera. Hair cell bundle counts were obtained in 19 predetermined locations (2500 μm^2 ^boxes) across the length of the saccule using Zeiss Axiovision software (Figure 1). This methodology to examine rostral-caudal shifts in saccular hair cell bundle density has been used previously for fishes [20,40], but the current study used more counting locations in order to obtain greater resolution of hair cell loss across the epithelia. The exact orientation of the counting boxes varied slightly between saccules because all saccules could not be mounted on the microscope slides at exactly the same angle. Nevertheless, the counting locations remained in the same area along the rostral-caudal axis and hair cell bundle counts remained consistent across saccules. Images of each saccule were merged in Photoshop CS2 (Version 9.0.2) in order to trace the perimeter of the damaged areas. Hair cell bundle counts across the width of the saccule were combined into seven regions along the lengthwise rostral-caudal axis of the saccule for statistical analysis (see Figure 1). In addition, the three counting locations within each rostral-caudal region were sorted by location across the width of the saccule (dorsal, central, or ventral). Region 4 was too narrow for three 2500 μm^2 ^counting locations, so only one central counting location was placed in this region.

### Statistical analysis

Hair cell bundle density in control fish varied along the length of the saccule (Figure 2). Thus, raw hair cell bundle counts in treatment fish were normalized by transformation to percent hair cell bundle loss as compared with mean control values. Preliminary analysis showed no significant differences between right and left saccules, so data for all saccules were pooled for further analysis. We used analysis of variance (ANOVA) to determine whether hair cell bundle loss varied significantly by region and frequency of tone exposure. Tukey's *post hoc *test was used to make pairwise comparisons between specific regions when significant main effects were found.

Hearing loss was quantified as a threshold shift (post-sound exposure threshold minus mean control threshold for a given frequency). An overall ANOVA was used to examine the effect of tone exposure on goldfish hearing thresholds. Similarly, ANOVAs were also used to test the main effects and interactions of treatment (tone) and frequency (hearing test frequency) on threshold shifts. Tukey's *post hoc *test was used to make pairwise comparisons between specific tone treatments and hearing frequencies when significant main effects were found.

The relationship between mean hair cell loss and TTS for each individual fish was analyzed using linear regression analysis. Means were calculated across all 19 hair cell counting locations for hair cell loss and all audiogram frequencies for TTS data. The three-dimensional surfaces shown in Figure 9 were constructed by distance-weighted least-squares smoothing of the original data, which imposes no *a priori *relationships between the variables. All statistical analysis was performed using SYSTAT Version 11 (Chicago, IL).

## Abbreviations

AEP: auditory evoked potentials; ANOVA: analysis of variance; DAPI: 4',6-diamidino-2-phenylindole; dB: decibel; FFT: Fast Fourier Transform; h: hours; Hz: hertz; LINTS: linear threshold shift; μPa: microPascal; MS-222: tricaine methanosulfonate; TS: torus semicircularis; TTS: temporary threshold shift: PTS: permanent threshold shift; RMS: root mean squared; SPD: sound pressure difference; SPL: sound pressure level

## Authors' contributions

MES conceived the study, did statistical analysis, and wrote the paper. JBS performed dissections of the goldfish saccules and editing of the manuscript. RRG did measurements and analysis of auditory sensitivity via auditory evoked potentials. BDR did microscope work and hair cell counts. MES, JBS, RRG, and BDR prepared the figures. All authors read and approved the final manuscript.
